# In vitro transfer of methicillin resistance determinants *mec*A from methicillin resistant *Staphylococcus aureus* (MRSA) to methicillin susceptible *Staphylococcus aureus* (MSSA)

**DOI:** 10.1186/s12866-017-0994-6

**Published:** 2017-04-04

**Authors:** Asinamai Athliamai Bitrus, Zakaria Zunita, Siti Khairani Bejo, Sarah Othman, Nur Adilah Ahmad Nadzir

**Affiliations:** 1 Faculty of Veterinary Medicine, Universiti Putra Malaysia, 43400 UPM Serdang, Malaysia; 2 Faculty of Biotechnology and Biomolecular Sciences, Universiti Putra Malaysia, 43400 UPM Serdang, Malaysia

**Keywords:** Antibiotics, Horizontal gene transfer, Methicillin, Resistance, *Staphylococcus aureus*

## Abstract

**Background:**

*Staphylococcus aureus* more than any other human pathogen is a better model for the study of the adaptive evolution of bacterial resistance to antibiotics, as it has demonstrated a remarkable ability in its response to new antibiotics. This study was designed to investigate the in vitro transfer of *mec*A gene from methicillin resistant *S. aureus* to methicillin susceptible *S. aureus*.

**Result:**

The recipient transconjugants were resistant to erythromycin, cefpodoxime and were *mec*A positive. PCR amplification of *mec*A after mix culture plating on Luria Bertani agar containing 100 μg/mL showed that 75% of the donor and 58.3% of the recipient transconjugants were *mec*A positive. Additionally, 61.5% of both the donor cells and recipient transconjugants were *mec*A positive, while 46.2% and 41.75% of both donor and recipient transconjugants were *mec*A positive on LB agar containing 50 μg/mL and 30 μg/mL respectively.

**Conclusion:**

In this study, the direction of transfer of phenotypic resistance as well as *mec*A was observed to have occurred from the donor to the recipient strains. This study affirmed the importance of horizontal transfer events in the dissemination of antibiotics resistance among different strains of MRSA.

## Background

*Staphylococcus aureus* is a good model better than any other human pathogen that exemplifies the successful adaptation to the therapeutic effect of antibiotics as it has demonstrated a unique ability in rapidly acquiring resistance to new antibiotics. It is one of the pathogens though extensively studied but yet, still surprises us with new and dynamic means of antibiotic resistance development.

The increase in antimicrobial resistance has coincided with the rate of widespread use of antibiotics. A strong selection pressure towards resistance among bacteria was promoted by the application of antibiotics in the fields of veterinary and human medicine as well as in animal husbandry [1]. It has been observed that the management of infectious disease is greatly threatened by the increase in the prevalence of antibiotic-resistant pathogens. Factors responsible for the development of resistance are associated with mobile genetic elements carrying genomic islands such as conjugative plasmids and transposons which are known to facilitate the transfer of resistance genes to other bacteria through horizontal gene transfer [2]. There is an increasing concern with regards to the emergence of MRSA as a common cause of hospital-acquired infections. This is because, majority of MRSA strains are multi-resistant, a feat achieved by the acquisition of extra resistance determinants such as conjugative plasmid carrying gentamicin resistance [2].

The site specific integration of the staphylococcus cassette chromosomes *mec* (*SCCmec*) into the genome of *S. aureus* at a region called the *SCCmec* insertion or attachment site is facilitated by a cassette chromosome recombinase (*ccr*) gene. [3, 4]. These recombinases function as dimers which in the case of *ccrAB,* facilitates the integration of *SCCmec* into the chromosome of *S. aureus.* This is achieved by attaching it to the core recognition site, one on the staphylococcal chromosome and the other on the *SCCmec* itself (*attB* and *attSCC*) [5]. The *attB* is a 15 base pair sequence found on the chromosomal end in the open reading frame of unknown origin (*orf*X) also known as the universal integration site for *SCCmec*. The integration of the *SCCmec* into the genome culminates into the formation of two hybrid site at either ends of the *SCCmec* dubbed the *attL* and the *attR* [5]. It is well-established that methicillin susceptible *S. aureus* (MSSA) became methicillin resistant *S. aureus* (MRSA) following the acquisition of genomic island carrying methicillin resistance determinant *mec*A [6]. However, the evolutionary origin as well as detail mechanism of transfer *mec*A is not fully understood [4, 7, 8].

In general, there are no studies available on the molecular investigation on antibiotic resistance transfer between MRSA and MSSA using isolates from human and animals and at different concentration of antibiotic marker. The increase in the emergence of highly pathogenic strains of methicillin resistant *S. aureus* and its impact on public health is becoming a major problem for prevention and control of MRSA. It is important therefore to investigate the mechanism of in vitro antibiotic resistance transfer and the role it plays in the emergence of highly resistant strains with a view to ensure effective prevention and control of *S. aureus* infection. This study was designed to investigate the in vitro transfer of methicillin resistance determinant *mec*A from MRSA to MSSA using mix liquid culture plating and PCR amplification of methicillin resistance gene *mec*A and *orf*X.

## Methods

### Bacterial strains and culture conditions

Six (6) methicillin resistant *S. aureus* (MRSA) and four (4) methicillin susceptible *S. aureus* (MSSA) strains isolated from humans (SH1, SH4 and SH8), animals (SDG2, SDG3, SDG4, SEQ1, SEQ5 and SCH4) and environment (SEV1) obtained from previous studies as reported by Aklilu et al. [9, 10] were used in this experiment. The isolates were collected from 2008 to 2011 from cats and dogs, DVM students and veterinary personnel and were identified as *S. aureus* using biochemical test and Staphylococcus identification kit, Staphytect plus(R) (Oxoid, UK) and Dry spot™ Staphytect plus (DR0100M, UK) according to the manufacturers recommendation before they were stored in cryobeads tubes at −80 °C as stock cultures. The cryobeads were thawed at room temperature before enrichment in tryptic soy broth and culturing onto a blood agar containing 5% horse. Presumptive isolates were reconfirmed as *S. aureus* by catalase and tube coagulase test as well as PCR amplification of thermostable nuclease gene (*nuc*). Phenotypic confirmation of MRSA was done by culturing on Oxacillin Resistance Screening Agar Base media (ORSAB) (Oxoid Basingstoke, UK) while genotypic confirmation was carried out by PCR amplification of methicillin resistance determinant *mec*A and *SCCmec* types. The recipient cells were also screened for the presence *orfX- SCCmec* integration site.

### Selection of donor and recipient cells

Donor and recipient cells were purposely selected based on their resistance profile, presence and absence of methicillin resistance determinant *mec*A, SCC*mec* types as well as availability of isolates with distinct antibiotic marker (Table 1). The donor cells were selected based on presence of *mec*A, type of *SCCmec* and susceptibility to tigecycline and levofloxacin but resistant to erythromycin and cefpodoxime. On the other hand, the recipients cells were *mec*A negative and resistant to tigecycline, levofloxacin but susceptible to erythromycin and cefpodoxime.

**Table 1:** Oligonucleotide sequence for the amplification of *mec*A, *orf*X and *SCCmec* types

S/N	Primer	Oligonucleotide sequence 5’to 3′	Product size	Annealing temperature	Reference
1	*mecA*F *mec*AR	ACTGCTATCCACCCTCAAAC	163 bp	57 °C/120 s	Noto. [27]; Mehrotra et al. [28]
		CTGGTGAAGTTGTAATCTG			
2	*Nuc-F*	GCG ATT GAT GG TGA TAC GGT T	276 bp	55 °C/120 s	Saiful et al. [29]
	*Nuc-R*	AGC CAA CGG TTG ACG AAC TAA AGC			
3	*Orf*X F	GAG AAA TAT TGG AAG CAA GCC	326 bp	54.6 °C/60s	Noto. [27]
	*OrfX* R	CGC ATA ATC TTA AAT GCT CTG			
4	SCC*mec*IIIFSCC*mec*IIIR	TTC TCA TTG ATG CTG AAG CC	280 bp	55 °C /60 s	Zhang et al. [30]
		GTG TAA TTT CTT TTG AAA GAT ATG G			
5	mecA F	5′-ACTGCTATCCACCCTCAAAC-3′	533 bp	55 °C/120 s	Saiful et al. [29]
	mecA R	5′-CTGGTGAAGTTGTAATCTGG-3′			

### Antibiotic resistance profile of bacterial strains

The antibiotic susceptibility profiles of the studied isolates were determined using disk-diffusion method as described by Bauer et al. [11]. The diameter of the zone of inhibition was measured using a digital Vernier caliper and interpreted according to the guidelines of Clinical Laboratory Standard Institute (CLSI) [12]. Susceptibility of the isolates were determined by testing each isolate against oxacillin 1 μg, cefoxitin 30 μg, cefpodoxime 10 μg, erythromycin 15 μg, amoxicillin 25 μg, tigecycline 15 μg, levofloxacin 1 μg and neomycin 10 μg.

### Transfer of antibiotic resistance in mixed liquid cultures

Fresh bacterial cultures were grown in Luria-Bertani broth (LB broth) at 30 °C for 12 h without shaking. Aliquot of 200 μL volume of each donor MRSA cells (resistant to erythromycin and cefpodoxime but susceptible to tigecycline and levofloxacin) and recipient MSSA cells (resistant to tigecycline and levofloxacin but susceptible to erythromycin and cefpodoxime) were adequately mixed in 1.5 mL microcentrifuge tube. The cultures were amplified with another 200 μL of LB broth and incubated for 6 h at 37 °C. Fifty microliter (50 μL) of the mixed cultures containing 10^5^ colony forming unit (CFU/mL) were drawn and plated on separate LB agar containing 100 μg/mL of erythromycin, tigecycline, cefpodoxime and levofloxacin. The procedure was repeated on LB agar containing 50 μg/mL and 30 μg/mL of the same antibiotics respectively and incubated for 24 h at 37 °C. After 24 h, colonies that grow on LB agar containing erythromycin or cefpodoxime were picked as the donors while those that grow on tigecycline or levofloxacin were picked as recipients’ transconjugants. The choices of these antibiotics were made on purpose so that the resistant colonies might represent the transconjugants where the transfer of methicillin resistance determinant *mec*A had taken place from the donor to recipient strains. Three to four colonies were picked from each of the plates and then inoculated in a freshly prepared LB broth and incubated overnight.

### DNA extraction

Genomic DNA was extracted from overnight fresh cultures by boiling method as described by Chen et al. [13]. A loopful suspension of overnight grown cultures was prepared in a 1.5 mL microcentrifuge tube containing 100 μL of sterile distilled water. The suspension was first incubated at room temperature for 5 min, and then heated in a dry bath at 96 °C for 10 min. The suspension was centrifuged at 13,000 rpm for 5 min, the supernatant was collected in a new microfuge tube and used as DNA template.

### Polymerase chain reaction (PCR) assay

PCR amplification of gene fragments of *mec*A (163 bp), *orfX* (356 bp) and *SCCmec* types was performed using specific primers and annealing cycling conditions as described in Table 1. All reactions were carried out in a thermal cycler (BIO-RAD) at initial denaturation temperature of 94 °C for 5 min, followed by 30–37 cycles of denaturation at 94 °C for 1 min, elongation at 72 °C for 1 min and final elongation at 72 °C for 5 min. The reaction was carried out in a 50 μL reaction volume which contained 5 μL genomic DNA, Toptaq master mix 25 μL (Qiagen), containing DNA polymerase, PCR Buffer (with 3 mM MgCl_2_), and 400 μM each dNTPs, 10× coral load 5 μL (Qiagen); 1 μL (0.1 μM) of each forward and reverse primer (Integrated DNA technologies, Singapore) and 13 μL of RNase free water (Qiagen).

### Agarose gel electrophoresis

Electrophoresis of the amplified PCR products was carried out in 2% agarose (Sigma-Aldrich) prepared in a 0.5X Tris-Borate EDTA (TBE) buffer. Ten microliter (10 μL) of PCR product each was loaded in a well of submerged gel. The PCR products were then subjected to electrophoresis at 80 V for 90 min. The gel was stained with Gel Red (Invitrogen) 2 μL/100 mL of agarose gel. The stained electrophoresed PCR products were visualized under the transilluminator UV-light using a gel documentation system alpha imager (BIO-RAD).

### Sequencing of *mec*A gene to validate the transfer events

**Fig. 1:**
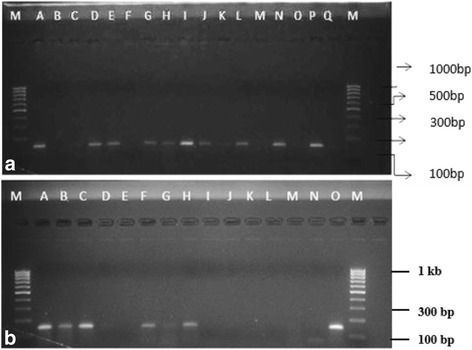
**a** Amplification of a 163 bp gene fragment of methicillin resistance determinants (*mec*A) fragments of MRSA. **b** Gel image showing PCR amplification of 276 bp thermostable nuclease (*nuc*) and 533 bp methicillin resistance determinant (*mec*A) gene fragment

Amplified chromosomal DNA containing the gene fragment of *mec*A (Fig. 1a and b) were sequenced using the BigDye^(R)^ Terminator v3.1 cycle sequencing kit (Applied Biosystems, USA). The electropherograms of the DNA sequence obtained from the eight isolates were checked for any ambiguity using BioEdit v7.0.9 [14]. The derived sequences were then subjected to multiple alignment sequence as in ClustalX (http://www.clustal.org/clustal2/) [15] using default parameters in order to obtain a consensus sequence. The consensus nucleotide fragments were then translated to protein using translating tool ExPasy (http://web.expasy.org/translate/) [16] and the open reading frame with was detected. This is to ensure that the *mec*A sequence were genuine and not due to errors arising from PCR which may introduce a shift in the frame and stop codons in the sequence.

## Result

### Antibiotic resistance profiles of donor, recipient, and transconjugants

All isolates were catalase, tube coagulase and *nuc* positive. All donor cells were susceptible to levofloxacin while two isolates (SH4 and SDG3) were resistant to tigecycline and cefpodoxime and in addition to oxacillin, cefoxitin, erythromycin, amoxicillin and neomycin (Table 2). The recipient cells were all susceptible to erythromycin, amoxicillin and levofloxacin with the exception of one isolate (SCH4). Additionally, three isolates (SDG4, SH8 and SEQ1) were resistant to tigecycline, two (SDG4 and SH8) for oxacillin and one isolate each (SEQ1, SH8) resistant to cefpodoxime, cefoxitin, and neomycin respectively (Table 2). The result of mix liquid culture plating on separate LB agar each containing antibiotics 100 μg/mL of erythromycin and cefpodoxime for selection of donor cells, tigecycline and levofloxacin for selection of recipient transconjugants, revealed two types of progeny cells; the donor cells which were resistant to erythromycin, cefpodoxime, oxacillin, cefoxitin, amoxicillin and neomycin and the recipient transconjugants were resistant to levofloxacin, tigecycline and neomycin. Similar result was obtained when the mix cultures were plated on LB agar containing 50 μg/mL and 30 μg/mL respectively. However, there was no growth when SEQ5 and SEQ1 were plated on LB agar containing 100 μg/mL of erythromycin and tigecycline but, growth was observed when the same cultures were plated on LB agar containing 50 μg/mL and 30 μg/mL erythromycin and tigecycline. But, the transconjugants were all *mec*A negative (Table 4).

**Table 2:** Antibiotic susceptibility profile and *mec*A status of donor and recipient cells

ID	Antibiotics Susceptibility profiles of donor cells								*mec*A	SCCmec
Donor cells	OX	CPD	FOX	E	N	AML	TGC	LEV		Type III
	1	10	30	15	10	25	15	1		
SH1	R	R	R	R	R	R	S	S	+	+
SH4	R	R	R	R	R	R	R	S	+	+
SDG2	R	S	R	R	R	R	S	S	+	+
SDG3	R	R	R	R	R	R	R	S	+	+
SEV1	R	S	R	R	R	R	S	S	+	+
SEQ5	R	S	S	R	R	R	S	S	+	+
Recipient cells	Antibiotics resistance profiles of recipient cells								*mec*A	*Orf*X
SDG4	R	S	S	S	S	S	R	S	-	+
SH8	R	R	S	S	S	S	R	S	-	+
SCH4	S	S	S	S	S	S	S	R	-	+
SEQ1	S	S	R	S	R	S	R	S	-	+

*SH* human isolate, *SDG* dog isolate, *SEV* environmental isolate, *SEQ* horse isolate, *SCH* chicken isolate, *R* resistance, *S* susceptible, *E15* Erythromycin, *CPD10* Cefpodoxime, *FOX30* Cefoxitin, *OX1* Oxacillin, *N10* Neomycin, *AML25* Amoxicillin, *TGC15* Tigecycline, *LEV1* Levofloxacin

### Methicillin resistance determinants (*mec*A) profiles of donor, recipient, and transconjugants

**Fig. 2:**
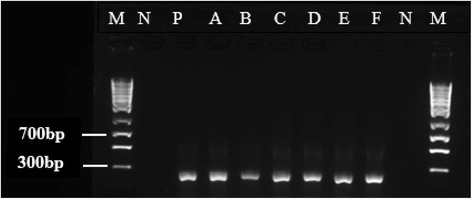
PCR amplification of a 356 bp gene fragment of the universal insertion site of SCC*mec* or open reading frame (*orf*X)

**Fig. 3:**
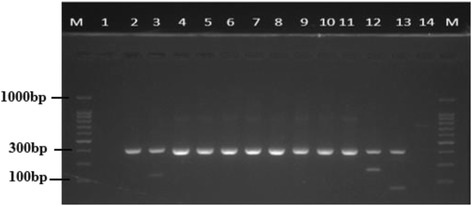
Electropherograph of a 280 bp gene fragment of Staphylococcus cassette chromosome *mec* type III

All donors used in this study were *mec*A positive and have the staphylococcal cassette chromosome *mec* a mobile genetic elements that harbors the *mec*A a determinant of methicillin resistance (Fig. 2). The presence of this structure in each donor isolate is to further confirm that the donor has the *mec*A gene. Additionally, the presence of *mec*A in the isolates helps to further categorize our isolates either as hospital acquired MRSA or community acquired MRSA. On the other hand, the recipient strains were *mec*A negative but positive for the *orfX* gene (Fig. 3). PCR amplification of methicillin resistance gene *mec*A after mix liquid plating on agar containing 100 μg/mL revealed that 75% (9) of the donor cells and 58.3% (7) of the recipient transconjugants cells were positive for *mec*A (Table 2). However, when cultures where plated on agar containing 50 μg/mL and 30 μg/mL respectively, 61.5% (8) of both the donor cells and recipient transconjugants were positive for *mec*A while only 46.2% (7) and 41.75%(5) of both donor cells and recipient transconjugants were *mec*A positive (Table 3).

**Table 3:** Methicillin resistance determinants *mec*A of donor and recipient transconjugants

SN	Cell type	Concentration of Antibiotic	*mec*A
1	Donor transconjugants	100 μg/mL	9/12 (75%)
	Recipient transconjugants	100 μg/mL	7/12 (58.3%)
2	Donor transconjugants	50 μg/mL	8/13 (61.5%)
	Recipient transconjugants	50 μg/mL	8/13 (61.5%)
3	Donor transconjugants	30 μg/mL	7/15 (46.2%)
	Recipient transconjugants	30 μg/mL	5/15 (33.33%)

### Validation of the *mec*A status of the progeny daughter cells

The methicillin resistance determinants *mec*A of progeny daughter cells after mixed liquid culture plating were determined using PCR assay. The amplified PCR products were then sequenced and analysed and the resultant consensus sequences shows 100% similarity in the nucleotide identity with the parent donor and recipient cells.

## Discussion

The rapid acquisition and worldwide dissemination of resistance determinants in MRSA is becoming a major veterinary and public health problem. This study was designed to investigate the in vitro transfer of antibiotic resistance from methicillin resistant *S. aureus* (MRSA) to a methicillin susceptible *S. aureus* (MSSA) using mix liquid culture plating. A number of studies have demonstrated the significance of horizontal transfer of resistance determinants from MRSA to MSSA as well as the role of PCR and selective antibiotics resistance markers in determining the direction of transfer [17–19].

In this study, growth observed on plates on LB agar containing 100 μg/mL of erythromycin-tigecycline, erythromycin-levofloxacin and cefpodoxime-levofloxacin each were considered as the transconjugants. Colonies on erythromycin and cefpodoxime plates were considered as donor cells while colonies that grow on tigecycline and levofloxacin plates were considered as recipient cells or transconjugants respectively. The antibiotic susceptibility profiles of the transconjugants when a dog isolate SDG2 serving as the donor strain was combined in a mix liquid culture plating with a human isolates SH8 serving as the recipient strain, revealed a transconjugants with a similar resistant profile to that of the donor strain as shown in (Table 4). The transconjugants were resistant to oxacillin, cefoxitin, erythromycin, amoxicillin, cefpodoxime, neomycin and tigecycline but sensitive to levofloxacin. Similar result was obtained when the same donor SDG2 was combined in a mix liquid culture plating with a different recipient strain SDG4 both isolated from dog. All the transconjugants were *mec*A positive, thus, indicating that transfer of methicillin resistance between the donors to recipient cell had occurred. Phenotypic transfer of cefoxitin, cefpodoxime, and erythromycin and amoxicillin resistance was also observed to have occurred from the donor strain to the recipient strains. Transfer of phenotypic resistance to tigecycline was also observed to have occurred from the recipient to the donor strain. This transfer was possible, because resistance to beta lactams and macrolides antibiotics are plasmid coded and transfer of resistance have been known to have occurred in the absence of detectable conjugative plasmid [4]. Majority of MRSA possesses the conjugative plasmid belonging to the PGO1/pSK41 lineage which carries an identical transfer gene and *ori*T sequences. These plasmids carry multiple antibiotic resistance genes which are known to be transferable between different staphylococcal species [20]. In addition, conjugative plasmid transfer and transposition of resistance gene have been reported in *S. aureus* [21]. Similar result was obtained when the mix cultures were plated on LB agar containing 50 μg/mL and 30 μg/mL of appropriate antibiotics however only one (SH8) of the two recipient transconjugants was positive for *mec*A gene. Even though MRSA does not have a detectable conjugative plasmids coding for the *tra* gene as in other bacteria which facilitate the transfer of resistance gene and subsequent emergence of strains with new form of resistance characteristics, evidence abounds that gene transfer events have been reported to have occurred [4, 22]. Therefore, this may be due to the transfer of phenotypic resistance as well as determinants of methicillin resistance *mec*A between the donor and recipient strains was possible.

**Table 4:** Antibiotic susceptibility profile and *mec*A status of donor, recipient and transconjugants

Cell type	ID		Antibiotic susceptibility profiles							*mec*A
		OX1	CPD10	FOX3O	E15	N10	AML25	TGC15	LEV	
Donor	SEQ5	R	S	S	R	R	R	S	S	+
Recipient	SCH4	S	S	S	S	S	S	S	R	−
(D)Transconjugants	E30	R	R	S	S	R	R	R	S	−
(R)Transconjugants	T30	R	S	S	S	R	R	R	S	−
Donor	SEQ5	R	S	S	R	R	R	S	S	+
Recipient cell	SEQ1	S	S	S	S	S	S	S	S	−
(D)Transconjugant	E30	S	I	S	S	R	R	R	S	−
(R)Transconjugant	T30	S	S	S	I	R	R	R	S	−
Donor cells	SEV1	R	S	R	R	R	R	S	S	+
Recipient cells	SEQ1	S	S	R	S	R	S	R	S	−
(D)Transconjugants	E30	R	R	R	R	R	R	R	S	−
(R)Transconjugants	T30	R	R	R	R	R	R	R	S	−
Donor	SEQ5	R	S	S	R	R	R	S	S	+
Recipient	SCH4	R	S	S	S	S	S	R	S	+
(D)Transconjugants	E50	ND	ND	ND	ND	ND	ND	ND	ND	ND
(R)Transconjugants	T50	S	S	S	S	R	R	R	S	−
Donor	SEQ5	R	S	S	R	R	R	S	S	+
Recipient cell	SEQ1	S	S	R	S	R	S	R	S	−
(D)Transconjugant	E50	ND	ND	ND	ND	ND	ND	ND	ND	ND
(R)Transconjugant	T50	R	I	S	I	R	R	R	S	+
Donor cells	SEV1	R	S	R	R	R	R	S	S	+
Recipient cells	SEQ1	S	S	R	S	R	S	R	S	−
(D)Transconjugants	E50	R	R	R	R	R	R	R	S	−
(R)Transconjugants	T50	R	R	R	R	R	R	R	S	−
Donor	SEQ5	R	S	S	R	R	R	S	S	+
Recipient	SCH4	R	S	S	S	S	S	R	S	+
(D)Transconjugants	E100	ND	ND	ND	ND	ND	ND	ND	ND	ND
(R)Transconjugants	T100	S	S	S	S	R	R	R	S	−
Donor	SEQ5	ND	ND	ND	ND	ND	ND	ND	ND	ND
Recipient cell	SEQ1	ND	ND	ND	ND	ND	ND	ND	ND	ND
(D)Transconjugant	E100	ND	ND	ND	ND	ND	ND	ND	ND	ND
(R)Transconjugant	T100	ND	ND	ND	ND	ND	ND	ND	ND	ND
Donor cells	SEV1	ND	ND	ND	ND	ND	ND	ND	ND	ND
Recipient cells	SEQ1	ND	ND	ND	ND	ND	ND	ND	ND	ND
(D)Transconjugants	E100	ND	ND	ND	ND	ND	ND	ND	ND	ND
(R)Transconjugants	T100	ND	ND	ND	ND	ND	ND	ND	ND	ND

*ND* Not determined, *SH* Staphylococcus human isolate, *SDG* staphylococcus dog isolate, *SCH* staphylococcus chicken isolate, *SEV* staphylococcus environmental isolate, *SEQ* staphylococcus equine/horse isolate, 100, 50, 30 concentration of antibiotics in μg/mL, *D* donors, *R* recipients, *D Transconjugants* donor transconjugants, *R Transconjugants* recipient transconjugants, *R* resistance, *S* susceptible, *I* intermediate resistance, *E15* erythromycin, *CPD10* cefpodoxime, *Lev1* levofloxacin, *TGC15* tigecycline

However, when another dog isolate SDG3 was used as the donor strain and combined with poultry isolate SCH4 resistant to levofloxacin as recipient strain in a mix liquid culture plating, the donor transconjugants had a similar profile with the parent donor strain which was resistant to oxacillin, cefoxitin, cefpodoxime, tigecycline, erythromycin, amoxicillin and neomycin while the recipient transconjugants was resistant to tigecycline, levofloxacin and neomycin a profile similar to the recipient parent strain. Similar results was observed when grown on agar containing 50 μg/mL and 30 μg/mL of appropriate antibiotics but, the recipient transconjugants were *mec*A negative. In each case phenotypic antibiotics resistance transfer was observed to have occurred in both direction, with the donor strain transferring resistance to neomycin and tigecycline while the recipient transferred levofloxacin resistance to the donor. Plasmid transfer of resistance to tetracycline, macrolide and quinolones have been reported to have occurred in *S. aureus* but, at a lower frequency [16, 23, 24]. The resistance to levofloxacin observed in donor transconjugants indicate that phenotypic transfer of resistance to levofloxacin have occurred from the recipient strain to the donor strain and so the direction of transfer can be said to be from recipient poultry isolate to donor poultry isolate while the inability of *mec*A transfer could be due restriction modification system inherent in the isolates [25] as transfer of resistant determinants is only possible between *S. aureus* of the same cluster even though the parent donor and recipient cells were both sourced from animal, yet can be different sequence type.

When a donor cell isolated from the environmental (SEV1) and SCH4 as recipient strains were combined, the transconjugants observed upon plating on agar containing 100 μg/mL and 50 μg/mL of the appropriate antibiotic resistance marker had profiles similar to both the donor and recipient parent cell and were *mec*A negative. Moreover, the transconjugants on plate containing 30 μg/mL antibiotics had resistance profile similar to the parent donor cells and were *mec*A positive (Table 4). The presence of *mec*A positive transconjugants when SCH4 was used as a recipient cell on agar containing 30 μg/mL of antibiotics could possibly be due to the fact that there was actual transfer of *mec*A from the parent donor cell to the parent recipient cells which was not observed when same isolates were plated on agar containing 100 and 50 μg/mL probably due to the higher concentration of the antibiotics. Thus, the rational for using different concentration of antibiotics, because each isolate had different minimum inhibitory concentration. This was however, not the case when the same donor (SEV1) was combined with a different recipient strain. It was observed that both the donor and recipient transconjugants were resistant to oxacillin, cefoxitin, cefpodoxime, erythromycin, amoxicillin and neomycin but sensitive to tigecycline and levofloxacin a profile similar to the parent donor strain. This however, shows that the transconjugants were more closely related to the parent donor than the recipient. The direction of transfer could not be established since both the donor and recipient transconjugants were susceptible to tigecycline and levofloxacin even though they were *mec*A positive. This was consistent with the findings of Sabet et al. [18] where he reported a transconjugants with resistance phenotype different from either the parent donor or recipient. From the results obtained it can be inferred that there was no antibiotic resistance transfer from MRSA to MSSA of animals’ origin and this could be due to the difference in strain or clonal class. The susceptibility of both the donor and recipient cells could be due to the concentration of the antibiotics in the two medium.

Likewise, when a horse isolate SEQ5 was used as the parent donor with a poultry isolate SCH4 and another horse isolate SEQ1 as recipients there was no growth observed on the LB agar containing the donor antibiotic resistant marker erythromycin. Similar result was also obtained when the concentration of the antibiotic marker was used at 50 μg/mL (Table 4). However, the recipient transconjugants had profiles similar to the parent recipient cells and all with the exception of one isolates were all *mec*A negative. However, this was not the case when the plating was done on 30 μg/mL of appropriate antibiotic resistance marker; the transconjugants observed were having profiles similar to either of the parent cells and were *mec*A negative. When the same donor was used with a dog isolate as recipient the transconjugants obtained were all *mec*A negative with profiles similar to both the parent and recipient cells. The inability of transfer to have occurred from the donor to the recipient strains could be the result of the presence of restriction system inherent in the bacteria, restriction system allows the integration of acquired gene to thrive only from bacteria of the same cluster [26]. The presence of an oxacillin positive *mec*A negative strain can occur due to the presence of the presence of a small colony variants of oxacillin resistant strains which occurred as a result of over production of penicillin binding protein rather than the acquisition of *mec*A from the donor strains. Additionally, resistance to oxacillin could also occur due to external factors such as the content of the medium which could slightly affect the outcome of the result as an increase in ±0.1 mm in the diameter of inhibition could alter the resistance profile of the isolate.

Furthermore on investigating antibiotic resistance transfer using two of the human isolates (SH1 and SH4) as donor cells with three isolates (SH8, SGD4 and SCH4) serving as recipients cells. The transconjugants obtained when SH1 was combined with SDG4, and SH1 with SH8 all had resistance profiles similar to the parent donor transconjugants and were all *mec*A positive. However, when SH4 was combined SCH4, the donor transconjugants had profiles similar to the parent donor cells and the recipient transconjugants also had profiles similar to the recipient parent cells and were *mec*A positive. Similar result was also obtained when mix culture was plated on agar containing 50 μg/mL and 30 μg/mL of antibiotic resistance marker (Table 5). This is in agreement with work of Khan et al. [17] where he demonstrated the in vitro transfer of erythromycin resistance from a clinical strain to poultry strain. The similarity observed in resistance profiles showed that the transconjugants were closely related to the donor strain than the recipient strain, however, the transfer of *mec*A shows that the direction of methicillin resistance transfer was from the human to animal isolate.

**Table 5:** Antibiotic susceptibility profiles and *mec*A status of donor and recipient transconjugants

Cell type	ID	Antibiotic profiles of transconjugants							*mec*A
		OX1	CPD10	FOX3O	E15	N10	AML25	TGC15	
(D)Transconjugant	1E	R	R	R	R	R	I	S	+
(R)Transconjugant	1 T	R	R	R	R	R	I	R	+
(D)Transconjugant	2E	R	R	R	R	R	R	R	+
(R)Transconjugant	2 T	R	R	R	R	R	I	S	+
(D)Transconjugant	3E	R	R	R	R	R	R	S	+
(R)Transconjugant	3 T	R	R	R	R	R	R	S	−
(D)Transconjugant	4E	R	R	R	R	R	I	R	+
(R)Transconjugant	4 T	S	S	S	S	S	R	R	−
(D)Transconjugant	5E	R	R	R	R	R	S	S	+
(R)Transconjugant	5 T	R	R	R	R	R	R	R	+

*Transconjugants (A)* donor transconjugants, *Transconjugants (B)* recipient transconjugants, *E, T*, agar concentration of erythromycin and tigecycline in μg/mL, *D* donors, *R* recipients, *D R* resistance, *S* susceptible, *I* intermediate resistance

## Conclusion

The use of PCR and antibiotic markers have enable us to determine the direction of transfer of resistance between MRSA and MSSA, however, the different concentration of the antibiotics used did not significantly affect the rate of transfer, as the difference in *mec*A status of the recipient transconjugants were not much. In this study, it was established that transfer of methicillin resistance determinants *mec*A have occurred from MRSA to MSSA at different concentration of antibiotic resistance marker.

## Abbreviations

MRSA: Methicillin resistant *Staphylococcus aureus*
MSSA: Methicillin susceptible *Staphylococcus aureus*
PCR: Polymerase chain reaction
LB: Luria-Bertini
OrfX: Open reading frame of unknown origin
SCC*mec*: Staphylococcal cassette chromosome *mec*
UV: Ultra violet
CFU: colony forming unit
DNA: Deoxyribonucleic acid
CLSI: Clinical laboratory standard institute
ORSAB: Oxacillin resistance screening agar base

## References

[1] Witte W (2000). Ecological impact of antibiotic use in animals on different complex microflora: environment. Int J Antimicrob Agents.

[2] Ohlsen K, Ternes T, Werner G, Wallner U, Löffler D, Ziebuhr W, Witte W, Hacker J (2003). Impact of antibiotics on conjugational resistance gene transfer in *Staphylococcus aureus* in sewage. Environ Microbiol.

[3] Berger-Bächi B, Rohrer S (2002). Factors influencing methicillin resistance in staphylococci. Arch Microbiol.

[4] Barlow M. What antimicrobial resistance has taught us about horizontal gene transfer. In: Horizontal Gene Transfer. Springer; 2009;397–411. doi:10.1007/978-1-60327-853-9. 10.1007/978-1-60327-853-9_23 19271198

[5] Wang L, Archer GL (2010). Roles of CcrA and CcrB in excision and integration of staphylococcal cassette chromosome *mec*, a *Staphylococcus aureus* genomic island. J Bacteriol.

[6] Chongtrakool P, Ito T, Ma XX, Kondo Y, Trakulsomboon S, Tiensasitorn C, Jamklang M, Chavalit T, Song J-H, Hiramatsu K (2006). Staphylococcal cassette chromosome mec (SCCmec) typing of methicillin-resistant *Staphylococcus aureus* strains isolated in 11 Asian countries: a proposal for a new nomenclature for SCC*mec* elements. Antimicrob Agents Chemother.

[7] Chambers HF (1997). Methicillin resistance in staphylococci: molecular and biochemical basis and clinical implications. Clin Microbiol Rev.

[8] Hanssen AM, Ericson Sollid JU (2006). SCC*mec* in staphylococci: genes on the move. FEMS Immunol Med Microbiol.

[9] Aklilu E, Zunita Z, Hassan L, Cheng CH (2013). Molecular epidemiology of methicillin-resistant *Staphylococcus aureus* (MRSA) among veterinary students and personnel at a veterinary hospital in Malaysia. Vet Microbiol.

[10] Aklilu E, Zunita Z, Hassan L, Chen H (2010). Phenotypic and genotypic characterization of methicillin-resistant *Staphylococcus aureus* (MRSA) isolated from dogs and cats at university veterinary hospital Universiti Putra Malaysia. Trop Biomed.

[11] Bauer A, Kirby W, Sherris JC, Turck M. Antibiotic susceptibility testing by a standardized single disk method. Am J Clin Pathol. 1966;45(4):493. 5325707

[12] Clinical and Institute Laboratory Standards (CLSI). Performance standards for antimicrobial disc susceptibility tests; Approved Standard-11th Ed, M2-A9. Wayne, PA, USA. 2014.

[13] Chen L, Mediavilla JR, Oliveira DC, Willey BM, de Lencastre H, Kreiswirth BN (2009). Multiplex real-time PCR for rapid staphylococcal cassette chromosome *mec* typing. J Clin Microbiol.

[14] Tom H (2013). Biological sequence allignment editor for win 95/98/NT/2A/XP/7.

[15] Thompson JD, Gibson TJ, Plewniak F, Jeanmougin F, Higgins DG (1997). The Clustal_X windows interface: flexible strategies for multiple sequence allignment aided by quality analysis tool. Nucleic Acid Research.

[16] Gaisteiger E, Gattiker A, Hoogland C, Ivanyi I, Appel RD, Bairoch A (2003). ExPASy: the proteomics server for depth protein knowledge and analysis. Nucleic Acid Research.

[17] Khan SA, Nawaz MS, Khan AA, Cerniglia CE (2000). Transfer of erythromycin resistance from poultry to human clinical strains of *Staphylococcus aureus*. J Clin Microbiol.

[18] Sabet NS, Subramaniam G, Navaratnam P, Sekaran SD (2014). In vitro mecA gene transfer among *Staphylococcus aureus* in Malaysian clinical isolates. Afr J Biotechnol.

[19] Wielders C, Vriens M, Brisse S, de Graaf-Miltenburg L, Troelstra A, Fleer A, Schmitz F, Verhoef J, Fluit A (2001). Evidence for in-vivo transfer of *mec*A DNA between strains of *Staphylococcus aureus*. Lancet.

[20] Ray M, Boundy S, Archer G. Transfer of the methicillin resistance genomic island among staphylococci by conjugation. Mol Microbiol 2016. 10.1111/mmi.13340 PMC4889012 26822382

[21] Edwards JS, Betts L, Frazier ML, Pollet RM, Kwong SM, Walton WG, Ballentine WK, Huang JJ, Habibi S, Del Campo M (2013). Molecular basis of antibiotic multiresistance transfer in *Staphylococcus aureus*. Proc Natl Acad Sci.

[22] Forbes BA, Schaberg DR (1983). Transfer of resistance plasmids from *Staphylococcus epidermidis* to *Staphylococcus aureus*: evidence for conjugative exchange of resistance. J Bacteriol.

[23] Roberts MC (2005). Update on acquired tetracycline resistance genes. FEMS Microbiol Lett.

[24] Doškař J, Pantůček R, Růžičková V, Sedláček I. Molecular Diagnostics of *Staphylococcus aureus*. In: Detection of Bacteria, Viruses, Parasites and Fungi. Springer; 2010;139–184. doi:10.1007/978-90-481-8544-3_7.

[25] Lindsay JA (2014). Evolution of *Staphylococcus aureus* and MRSA during outbreaks. Infect Genet Evol.

[26] Huddleston JR (2014). Horizontal gene transfer in the human gastrointestinal tract: potential spread of antibiotic resistance genes. Infect Drug Resist.

[27] Noto MJ, Kreiswirth BN, Monk AB, Archer GL (2008). Gene acquisition at the insertion site for SCC*mec*, the genomic island conferring methicillin resistance in *Staphylococcus aureus*. J Bacteriol.

[28] Mehrotra M, Wang G, Johnson WM (2000). Multiplex PCR for Detection of genes for *Staphylococcus aureus* Enterotoxins, Exfoliative toxins, toxic shock syndrome toxin 1, and Methicillin resistance. J Clin Microbiol.

[29] Saiful A, Mastura M, Zarizal S, Mazurah M, Shuhaimi M, Ali A (2006). Detection of methicillin-resistant *Staphylococcus aureus* using *mec*A/*nuc* genes and antibiotic susceptibility profile of Malaysian clinical isolates. World J Microbiol Biotechnol.

[30] Zhang K, McClure J-A, Conly JM (2012). Enhanced multiplex PCR assay for typing of staphylococcal cassette chromosome *mec* types I to V in methicillin-resistant *Staphylococcus aureus*. Mol Cell Probes.

